# Diet selectivity in a terrestrial forest invertebrate, the Auckland tree wētā, across three habitat zones

**DOI:** 10.1002/ece3.3763

**Published:** 2018-02-01

**Authors:** Matthew B. G. J. Brown, Chrissen E. C. Gemmill, Steven Miller, Priscilla M. Wehi

**Affiliations:** ^1^ School of Science University of Waikato Hamilton New Zealand; ^2^ Department of Mathematics and Statistics School of Computing and Mathematical Sciences University of Waikato Hamilton New Zealand; ^3^ Centre for Sustainability University of Otago and Manaaki Whenua Landcare Research Dunedin New Zealand

**Keywords:** feeding behavior, *Hemideina thoracica*, insect, lipids, nutrition, protein, selective foraging

## Abstract

Insects are important but overlooked components of forest ecosystems in New Zealand. For many insect species, information on foraging patterns and trophic relationships is lacking. We examined diet composition and selectivity in a large‐bodied insect, the Auckland tree wētā *Hemideina thoracica*, in three habitat zones in a lowland New Zealand forest. We asked whether *H. thoracica* selectively forage from available plant food sources, and whether these choices were lipid‐rich compared to nonpreferred available plants. We also identified the proportion of invertebrates in their frass as a proxy for omnivory. From reconnaissance plot sampling, together with fecal fragment analysis, we report that more than 93% of individual tree wētā had eaten invertebrates before capture. Additionally, wētā in the highest elevation hillslope habitat zone consumed significantly fewer species of plants on average than wētā on the low‐elevation terrace habitat. Upper hillslope wētā also had the highest average number of invertebrate fragments in their frass, significantly more than wētā in the low‐elevation terrace habitat zone. Wētā showed high variability in the consumption of fruit and seeds across all habitat zones. Generally, we did not observe diet differences between the sexes (although it appears that male wētā in the mid‐hillslope habitat ate fruits and seeds more voraciously than females), suggesting that the sexes have similar niche breadths and display similar degrees of omnivorous behavior. Extraction of leaf lipids demonstrated a range of lipid content values in available plants, and Ivlev's Electivity Index indicated that plant species which demonstrated high electivity tended to have higher concentrations of lipids in their leaves. Our findings indicate that *H. thoracica* forage omnivorously and selectively, and hence play multiple roles in native ecosystems and food webs.

## 1 INTRODUCTION

Feeding is ultimately about securing nutrients for growth and reproduction (Lee, Cory, Wilson, Raubenheimer, & Simpson, 2006; Simpson, Sibly, Lee, Behmer, & Raubenheimer, 2004; Slansky & Wheeler, 1991). Many animals regulate their consumption of food sources via a combination of pre‐ and post‐ingestion mechanisms to meet their developmental and physiological requirements (Behmer, 2009; Mayntz, Raubenheimer, Salomon, Toft, & Simpson, 2005; Schiff, Waldbauer, & Friedman, 1988). Resources may be used disproportionately to their availability. In these scenarios, it benefits animals to forage selectively. Leaf‐eating generalists therefore feed on a range of plant species, switching species during and between individual bouts of foraging, in order to maximize their nutrient intake, while minimizing the consumption of toxic plant metabolites (Behmer, Simpson, & Raubenheimer, 2002; Pulliam, 1975; Rapport, 1980; Raubenheimer, 1992; Singer, Bernays, & Carriere, 2002).

Nitrogen (N) is frequently a limiting factor for animals in the wild (Denno & Fagan, 2003; Lee‐Yaw et al., 2016; Nersesian, Banks, & McArthur, 2011; Nersesian, Banks, Simpson, & McArthur, 2012), including herbivorous Orthoptera (Joern & Behmer, 1997). Multiple demographic traits such as growth rate, survival, and fecundity are positively associated with N consumption (Joern & Behmer, 1997), and many terrestrial arthropods have evolved foraging strategies that enhance nitrogen intake, such as feeding on invertebrates that are high in N‐rich molecules (e.g., Denno & Fagan, 2003). The consumption of N‐rich food sources can also benefit animals by offsetting the energetic cost of metabolising toxic plant metabolites, enabling the consumer to eat more leaf materials before toxin loading forces them to stop eating, or switch plants (Nersesian et al., 2011, 2012; Raubenheimer, 1992; Raubenheimer & Simpson, 1990). Nonetheless, animals may also be limited by macronutrients or lipids that are energy sources for metabolism, growth and reproduction, as well as contributing to other functions such as cuticular water loss (e.g., Beenakkers, Van der Horst, & Van Marrewijk, 1981). Lipid limitation can result in predators consuming lipid‐rich plant‐based foods, and feeding low in trophic food webs (Wilder, Norris, Lee, Raubenheimer, & Simpson, 2013). Experimental studies have shown that multiple groups of predatory and omnivorous arthropods are more limited by lipids than nitrogen, including beetles and spiders (Mayntz & Toft, 2001; Mayntz et al., 2005; Raubenheimer, Mayntz, Simpson, & Toft, 2007).

New Zealand tree wētā (pūtangatanga, *Hemideina* spp., Orthoptera: Anostostomatidae) are generalist insects that are widespread and abundant in lowland podocarp broadleaved forests of New Zealand, as well as other habitats. However, little is known about their feeding ecology. In all, there are seven species within this endemic genus. Adults are large (2–8 g; Griffin, 2011), and long‐lived, with some species living for 2–3 breeding seasons after reaching sexual maturity, which may take over a year (Leisnham, Cameron, & Jemieson, 2003). Flightless and nocturnal, they are a favoured prey for native New Zealand birds (e.g., ruru/morepork, *Ninox novaeseelandiae* Gmelin, 1788; kiwi, *Apteryx* spp. Shaw, 1813; kākā, *Nestor meridionalis* Gmelin, 1788), as well as introduced mammalian predators such as ship rats (*Rattus rattus* Linnaeus, 1758; Ruscoe & Murphy, 2005), mustelids such as ferrets (*Mustela furo* Linnaeus, 1758; King & Murphy, 2005), and hedgehogs (*Erinaceus europaeus* Linnaeus, 1758; Jones & Sanders, 2005).

Tree wētā are important forest consumers, feeding on the leaves of a variety of native and non‐native plant species (Dewhurst, 2012; Griffin, Morgan‐Richards, & Trewick, 2011), but many aspects of their diet and foraging are not yet quantified, including the importance of omnivorous feeding to gain N‐rich foods (Wehi, Raubenheimer, & Morgan‐Richards, 2013). Tree wētā populations in secondary or modified forests have been estimated to consume between 7.3 and 182.5 kg of leaf materials ha^−1^ year^−1^, at densities ranging from 180 to 5,000 wētā/ha (Moller, 1985; Townsend, Brown, Stringer, & Potter, 1997). However, because tree wētā are cryptic and nocturnal, it is difficult to observe foraging directly. Wehi and Hicks (2010) concluded that *Hemideina thoracica* White, 1842, in urban forest fragments were primarily herbivorous, based on stable isotope analysis. Dewhurst (2012) used frass analysis for *H. thoracica* and *Hemideina crassidens* Blanchard, 1851, collected from an intact lowland forest in the lower North Island to identify fragments from 29 different plant species.

Tree wētā are also predators, although they are not obligate omnivores (Griffin et al., 2011). Wētā from this genus in the subalpine zone (*Hemideina maori* Pictet and Saussure, 1891) are known to prey on invertebrates (Lodge, 2000; Wilson, 2004; Wilson & Jamieson, 2005) but may also potentially target lipid‐rich plants (Joyce, 2002; Lodge, 2000; Wilson, 2004; Wilson & Jamieson, 2005). Dewhurst (2012) also noted the presence of invertebrate fragments in tree wētā frass from a lowland North Island forest, but the frequency and abundance of these invertebrate fragments relative to plant fragments is unknown. Dewhurst (2012) hypothesized that nitrogen might be a limited nutrient, as with many insects, and performed captive feeding trials to determine whether tree wētā preferentially selected leaves of plant species with high concentrations of nitrogen. However, results were equivocal, and overall, N concentration did not successfully predict relative preference. This suggests that there are likely other factors that determine the foraging behavior. We do not yet have any evidence for selective feeding patterns in other species of tree wētā in lowland forest habitat, such as *H. thoracica*.

Tree wētā may also be seed dispersers: one 50‐year‐old record documents the widely distributed Auckland tree wētā *H. thoracica*, browsing on kauri (*Agathis australis*) seed on the forest floor (Mirams, 1956). Frugivory has been recorded in captive trials with other tree wētā species. Duthie, Gibbs, and Burns (2006) fed fleshy fruits of 19 native species to captive *H. crassidens*. Of these, seeds from five plant species passed through wētā intact, and had a higher germination rate than seeds not ingested by wētā, suggesting that wētā may be seed dispersers. Nonetheless, eating fruits and seeds is poorly quantified in forest environments.

The aim of this study was to investigate diet composition and breadth in wild Auckland tree wētā *H. thoracica* (henceforth “wētā”)*,* in three habitat zones across an environmental gradient within a native lowland podocarp broadleaved forest. We assessed polyphagy and estimated the proportion of invertebrates as well as fruits and seeds in the diet of wētā to assess degree of omnivory. Finally, we asked whether *H. thoracica* feed preferentially on lipid‐rich plant sources and asked whether the lipid concentration of plants affected the probability that wētā chose to eat those plants, after accounting for plant availability. Our findings provide new insights on the foraging ecology of *H. thoracica* in lowland forests.

## 2 MATERIALS AND METHODS

### 2.1 Study site

**Figure 1:**
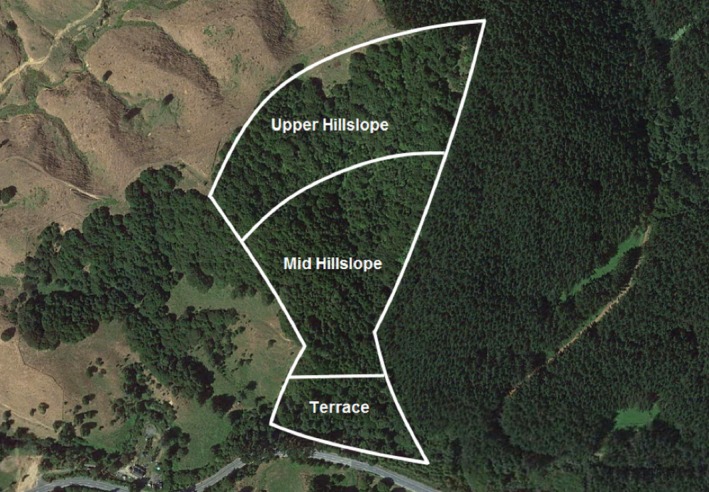
The study site, located on the northern edge of the Waingaro Forest Reserve, Tainui Ecological Region, Raglan Ecological District, North Island, New Zealand. The three topographical zones of terrace (T), mid‐hillslope (M), and upper hillslope (U) are shown (Image from Google Earth, 2013)

The study was conducted in a mixed podocarp broadleaf forest, dominated by kahikatea (*Dacrycarpus dacrydioides*) and tōtara (*Podocarpus totara*) in the Waingaro Forest Reserve (WFR), Tainui Ecological Region, Raglan Ecological District, North Island, New Zealand (37°40′25.74″S and 174°58′21.33″E) (Figure 1). The study site forms a 7.64‐ha catchment and is drained by a first‐order stream. The site faces west, and the slope has a moderate‐to‐steep aspect with mean elevation ranging from 47.1 to 127.5 m. The study site is divided into three distinct habitat zones: the lower terrace (T), the mid‐hillslope (M), and the upper hillslope (U). Drainage and exposure both increase from the terrace (the most poorly drained and the lowest amount of exposure) up to the hillcrest (the most well drained and the highest level of exposure), and the floral community changes along this gradient between zones, in response to the changes in these two variables.

### 2.2 Vegetation sampling and wētā collection

We compared counts of identified plant fragments against the relative ground cover of those plants following established methods (Bekhuis, De Jong, & Prins, 2008; Dewhurst, 2012; Wilson, 2004). To estimate the relative ground cover, a surrogate for food availability, of all plant species in each of three habitat zones, we conducted reconnaissance (recce) plot sampling with 10 evenly distributed 12 × 12 m recce plots in each zone. We recorded the area of ground covered by each species in each plot, using six standard cover abundance classes (<1%, 1%–5%, 6%–25%, 26%–50%, 51%–75%, and 76%–100%) at each of the six vertical height classes described in Hurst and Allen (2007). We used these data to determine the mean ground cover (%) of each identified plant species at each tier, and the tier with the highest mean coverage was used as the overall ground cover value for each species. Due to the effect of horizontally overlapping branches, the combined coverage of all plant species can be greater than 100%. Therefore, for analysis, the coverage for each species was adjusted to a percentage of the total coverage, bringing the total coverage in each habitat zone to 100%. Mean total cover and adjusted cover of all plant species, in all habitat zones, is provided in Appendix S1.

We carefully searched each plot for *H. thoracica*, which we collected from inside dead branches, fallen logs, and tree cavities. We recorded plot number, sex, host tree species, and habitat zone for each wētā (Table 1). Each wētā was placed alone into a separate 2‐L labeled covered container, with a substrate of moistened paper towels, and housed at the animal containment facilities at the University of Waikato, Hamilton, North Island, New Zealand. Captive wētā were fasted for 96 hr or until they stopped producing frass. Every frass pellet from every wētā was collected into an individual labeled 5‐ml falcon tube and stored at −20°C until processing.

**Table 1:** Number of Auckland tree wētā (*Hemideina thoracica*) captured on host trees in each habitat zone and mean number of plant species eaten by each wētā

Zone	Total # Captured	*Ls*	*Pt*	*Ke*	*Dc*	Mean Species Eaten
T	18	13	5	0	0	7.06
M	13	3	1	7	2	4.77
U	14	0	3	11	0	3.21

T, terrace; M, mid‐hillslope; U, upper hillslope; Host plants: *Ls*,* Ligustrum sinense*;* Pt*,* Podocarpus totara*;* Ke*,* Kunzia ericoides*;* Dc*,* Dacrydium cupressinum*.

### 2.3 Fragment library

To identify the processed fecal fragments, we first constructed a photographic reference library of plant fragments. We collected leaf materials from plant species present in the recce plots, and where available, flowers, fruits, and seeds. The slurry was spread onto standard glass microscope slides and stained with fuchsin gel following methods adapted from Lodge (2000), Joyce (2002), and Wilson (2004), and then photographed using a Leica DMRE light microscope. Plant species observed at the site, but which were not present in any recce plots, were not included. Morphological features of leaves that were most useful for successfully identifying fragments to the level of species were the size, shape, and arrangement of the stomata and epidermal cells, and features of the trichomes (Figure S1a–f in Appendix S2). Because the stomata and epidermal patterns of *Melicytus micranthus* and *M. ramiflorus* appear almost identical under magnification, they were identified only to the rank of genus. Six additional tissue types were recognized in the frass, but could not be identified (Figure S2, Appendix S2).

### 2.4 Frass composition

We next compared counts of fecal fragments belonging to different plant species against the relative ground cover of those plants following established methods (Bekhuis et al., 2008; Dewhurst, 2012; Wilson, 2004). Although fragment analysis is considered an accurate method of identifying the diet of animals that are difficult to directly observe feeding (Wilson, 2004), using a mortar and pestle to process frass can potentially fracture existing fragments and increase the representation of some fragment types. In this work, the mouthparts and proventriculus of tree wētā themselves acted to reduce plant material into very small fragments, and we took care to ensure that plant fragments were separated out from the frass pellets, but with minimal further pulverizing. In the reference library, hand‐processed samples were typically much larger than fragments present in actual wētā frass samples (M. Brown, personal observation), acting as a further quality control for our method.

Each frass pellet was individually processed in the same manner as the plant material to make slides. All fragments on every slide were identified and counted. We grouped all frass fragments observed into three diet categories: plant species fragments observed (where the total number of plant species observed is referred to as “diet breadth”); fragments derived from fruit and seeds; and invertebrates. Invertebrate fragments typically included bits of the rigid outer layer of the exoskeleton, or exocuticle and the pliable, inner layer of the exoskeleton, or endocuticle (Figure S1g,h, Appendix S2), as well as antennal fragments, pieces of compound eyes, tarsal claws, and leg joints and segments.

We identified plant fragments at 400× magnification and recorded the presence of each identifiable plant species from the frass pellet. We calculated the percent contribution of each plant species to the total number of fragments identified, for each frass pellet for each wētā. To expedite counts, if 50 fragments of a particular taxon were counted in a single slide, those fragments would cease being counted in that slide; we estimated the total via extrapolation based on the proportion of the slide that remained to be viewed. Full counts for all fragments produced by all individuals, in each habitat zone, are provided in Appendix S3.

### 2.5 Lipid concentration

We assayed seven plant species that were well represented in the wētā frass and present at the site, as well as eight plant species that were common at the site but which were not observed in wētā frass fragments. Lipids were extracted from fresh leaves using the method of Hara and Radin (1978) as follows. Leaves were collected from in situ plants, with 10 individuals per plant species except *Prumnopitys taxifolia* (*n* = 9). Leaves were collected in the field and kept as cool as possible by placing on ice. Approximately 1 g of fresh leaf material was used, with the weight of the leaves recorded prior to storage at −30°C. Frozen leaf material was ground into a powder using a mortar and pestle with liquid nitrogen and then transferred into a labeled 50‐ml Falcon tube containing 8 ml of isopropanol heated to 80°C and then heated a further 10 min. Samples were then cooled to room temperature; 12 ml of hexane was added, and samples were vigorously shaken before placing in an orbital shaker at 200 rpm for 10 min. We used hexane as the solvent, as hexane bonds to lipids more effectively than solvents such as ethanol (Ferreira‐Diaz, Valente, & Abreu, 2003). Next, 10 ml of aqueous sodium sulfate (prepared from 1 g of the anhydrous salt and 15 ml of water) was added to each sample, which was vigorously shaken before placing in an orbital shaker at 200 rpm for 10 min. Samples were centrifuged at 3130 rcf in an Eppendorf 5810R centrifuge for 5 min. The top layer was then transferred to a pre‐weighed 15‐ml Falcon tube, using a funnel lined with filter paper to remove particulates. Hexane was evaporated using compressed air and gentle heat. The tube was weighed again to determine lipid content, where the change in mass is the amount of lipid present (expressed as mg of lipids g^−1^ of leaf material).

### 2.6 Statistical analysis

Statistical analyses were performed in R 3.4.0 (R Core Team, 2013) using package multcomp (Hothorn, Bretz, & Westfall, 2008). We investigated tree wētā diet using the three diet categories recorded above: plant species, fruit and seeds, and invertebrates. To account for variable frass output among wētā, we first divided the total number of fragments identified in each category by the number of pellets produced, to obtain average values in each category for each wētā. We used a Poisson rate model with a log link function to model the average values for each diet category, and corrected for overdispersion in the models for fruit and seeds, and invertebrates. We included sex, habitat zone, and their interaction in each initial model. However, sex and interaction effects were insignificant for all models, based on chi‐square tests of changes in residual deviance, so final models for each diet category included habitat zone only. We used Tukey's multiple comparisons tests to determine which habitat zones were different for each diet category.

We calculated Ivlev's Electivity Index (IEI; Ivlev, 1961) as a measure of utilization of plant tissues as food sources, in relation to the abundance or availability of plant species in the environment. In this case, we used percent ground cover as a measure of availability. We used the equation *E*_i_ = (*r*_i_ − *n*_i_)/(*r*_i_ + *n*_i_), where *E*_i_ = species electivity, *r*_i_ = the proportion of a species in the diet, and *n*_i_ = the proportion of that species available in the habitat. Values range from −1 to 1, −1 indicates total avoidance, 0 indicates than an item is taken in proportion to its abundance in the ecosystem, and 1 indicates total preference (Ivlev, 1961). We considered a value greater than 0.3 as a high value, and hence potentially high preference. Confidence intervals for Ivlev's Index were calculated following Strauss (1979). Selection for or against a plant species type was considered when the 95% CI were either above or below zero, respectively. The calculated values for the IEI are best understood in conjunction with our other data, as the proportion of a species in the diet was calculated as the number of fragments of that species found in the wētā's frass divided by the total number of fragments for all plants, insects, and fruit and seeds, rather than by weight.

Based on 9–10 samples from each of 15 plant species present at the site, seven of which had high IEI scores, and eight of which had low IEI scores, we used a linear mixed‐effects model to test for significantly different mean lipid concentrations between the two groups. To test whether the lipid concentration of plants significantly affected the probability that wētā choose to eat those plants, after accounting for plant availability, we used the mean lipid concentrations of these 15 species, and the respective ground cover percentages for these species in each habitat zone to construct a logistic model with a logit link function.

## 3 RESULTS

### 3.1 Wētā frass and fecal fragments

**Figure 2:**
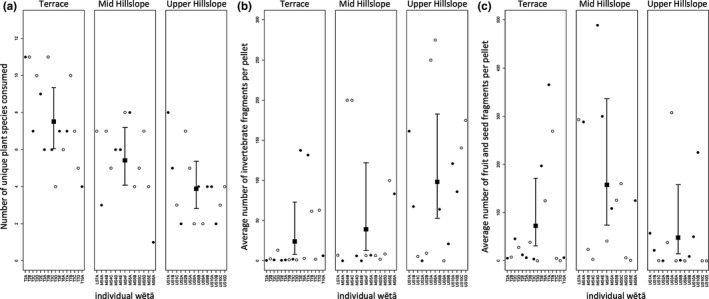
Wētā diet breadth across the three habitat zones, terrace, mid‐hillslope, upper hillslope for three different food categories (a–c). Individual wētā are represented in each column, with males represented by solid circles and females represented by open circles. (a) Average breadth of plant dietary items for each wētā. (b) Average number of invertebrate fragments per pellet per wētā. (c) Average number of fruit and seed fragments per pellet per wētā. Means over all wētā within a habitat zone and confidence intervals are indicated. Data were adjusted to account for a variable number of pellets for each wētā

Of 45 *H. thoracica* held in the animal house facilities, 43 produced frass over the 4‐day depuration period. The two wētā that did not produce any frass were therefore excluded from the analyses. In total, the 43 wētā produced 121 pellets. We identified a total of 49,952 fragments from plant material in the frass, from 18 different plant species representing 13 families (Appendix S3). Six additional unique leaf tissue types were documented, but could not be identified. After adjusting for the number of pellets produced, the mean number of plant species identified in frass cuticular fragments differed significantly between habitat zones (*n* = 43, χ^2^ = 14.708, *df* = 2, *p* < .001). However, there was considerable variance between individuals (Figure 2a). We did not observe a significant difference between average breadth of plant species eaten for the terrace and mid‐hillslope groups. We estimate the average breadth of plant species eaten of the mid‐hillslope group to be 0.83 times the average breadth of the terrace group (95% confidence interval 0.57–1.22 times, *p* = .4989, using Tukey's method for multiple comparisons). The difference in average breadth for plant species eaten between the upper hillslope and mid‐hillslope groups was also not significant. We estimate the upper hillslope group has an average breadth 0.66 times the average breadth of the mid‐hillslope group (95% confidence interval 0.43–1.01 times, *p* = .0599). The upper hillslope group had a significantly lower average breadth of plant species eaten than the terrace group. We estimate the upper hillslope group has an average breadth 0.55 times the average breadth of the terrace group (95% confidence interval 0.37–0.80 times, *p* < .001).

More than 93% of individual wētā had eaten other invertebrates before capture (Appendix S2 and Figure 2b), based on our total observations of invertebrate fragments in frass; only three tree wētā individuals had no invertebrate fragments identified in their frass. We detected a significant difference in the mean number of invertebrate fragments per pellet by habitat (*n* = 43, χ^2^ = 8.907, *df* = 2, *p* = .012, corrected for overdispersion). The upper hillslope group had a significantly higher mean number of invertebrate fragments per pellet than the terrace group. We estimate the upper hillslope group has a mean number of invertebrate fragments per pellet 4.10 times the mean number of invertebrate fragments per pellet of the terrace group (95% confidence interval 1.18–14.27 times, *p* = .022, using Tukey's method for multiple comparisons). In contrast, there were no apparent differences between the average number of invertebrate fragments per pellet for the mid‐hillslope and terrace groups (estimate = 1.63 times, 95% confidence interval 0.34–7.74 times, *p* = .739) nor the mid‐hillslope and upper hillslope groups (estimate = 2.51 times, 95% confidence interval 0.71–8.91 times, *p* = .203).

More than 86% of individual wētā had eaten fruit and seed before capture (Appendix S3), based on observations of the total frass, and fruit and seeds constituted 18.45% of all identified fragments. There was nevertheless a large variance in the number of fruit and seed fragments observed in the frass of individual wētā (Figure 2c). For example, three males in the mid‐slope habitat ate fruit and seeds voraciously and produced large quantities of these fragments. When we compared wētā from the three habitat zones, we did not detect any significant differences in the mean number of fruit and seed fragments in wētā frass (*n* = 43, χ^2^ = 4.903, *df* = 2, *p* = .086, corrected for overdispersion).

The majority of plant species recorded were found in the frass less often than expected based on adjusted percent cover (Appendix S1). Plants with highly positive IEI values, which hence appear to be eaten more than expected, were *Hymenophyllum flabellatum* (0.87*,* upper hillslope [U]), *D. dacrydioides* (0.52, terrace [T])*, Pennantia corymbosa* (0.3, T), and *Carpodetus serratus* (0.4, U; Appendix S1). Three taxa were eaten more than expected in two habitat zones: *P. totara* (0.34 mid‐hillslope [M]; 0.21 U)*, Kunzea ericoides* (0.48 M; 0.34 U), and *Melicytus* sp. (0.39 T; 0.27 M).

**Figure 3:**
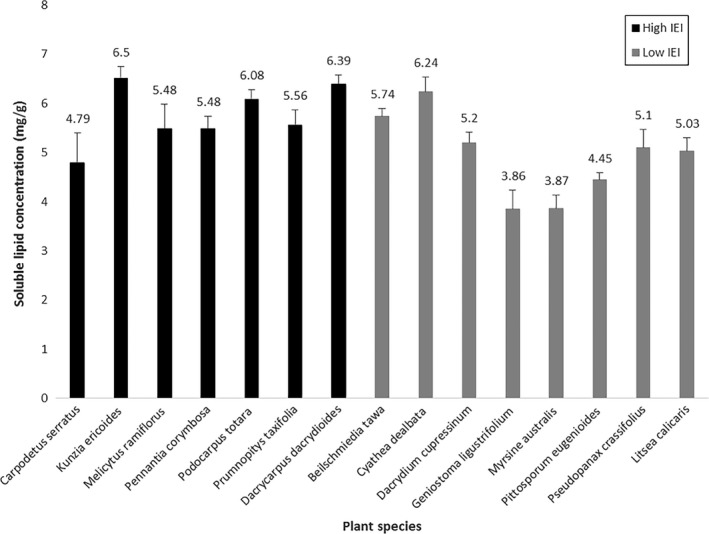
Concentration of lipids (mg/g) in the leaves of seven plants with high electivity (eaten), and eight plants that were common but had low electivity (not eaten) in the three habitats zones surveyed at Waingaro Forest Reserve. Standard errors are indicated

Mean concentration of lipids varied considerably between plants with high electivity (5.76 ± *SE* 0.281 mg/g) and those present but not eaten (4.93 ± *SE* 0.263 mg/g) (Figure 3). Three separate species in the preferred group (*K. ericoides*,* D. dacrydioides,* and *P. totara*) had soluble lipid concentrations that were comparable to, or greater than, the highest‐ranking plant in the noneaten group (*Cyathea dealbata*). The change in deviance of the models with and without the electivity term was significantly different from zero (χ^2^ = 4.494, *df* = 1, *p* = .034), suggesting a significant difference in mean lipid concentrations between the high and low electivity groups. A 95% profile confidence interval for the difference in mean lipid concentration between the high and low electivity groups is 0.07–1.57 mg/g.

When modeling the probability that a wētā eats a particular species, and after accounting for overdispersion, we found no significant difference between a model that allowed for interactions between the habitat zones and each of the mean lipid concentration of a plant species and the percentage ground cover of the plant species in each zone and a reduced model that included an effect for mean lipid concentration alone (*n* = 33, χ^2^ = 37.73, *df* = 7, *p* = .607, corrected for overdispersion). As the mean lipid concentration of a plant species increased, there was a significant increase in the odds that a wētā would eat that plant (*t* = 2.099, *df* = 31, *p* = .044, 95% confidence interval for multiplicative factor for the odds of eating a plant species = 1.10–5.13 times for each additional 1 mg/g in lipid concentration).

## 4 DISCUSSION

Foraging insects are grouped based on their degree of dietary specialization, with categories ranging from monophagous to polyphagous (Ali & Agrawal, 2012). In this study, the data show that *H. thoracica* consumes leaves, fruits, and seeds from a variety of native plants, representing a diverse range of plant families. In total, 18 species of plants, from 13 families including ferns, gymnosperms, and angiosperms, were positively identified in the frass. Despite this polyphagy, the variance among individual wētā in the number of species identified from plant tissues in individual frass (Figure 2a) suggests that individuals within a population can adopt specialist or generalist foraging strategies, as has been observed in other species (see, e.g., Bolnick et al., 2002), although it could also simply reflect differing opportunities for omnivory. In addition, the mean number of plant species present in the frass of upper hillslope *H. thoracica* was significantly less than terrace *H. thoracica*, indicating that diet breadth can vary with habitat, but further data are required to explore both prey invertebrate and habitat patterns.

We recorded invertebrate fragments in the frass of 93% of individuals (Appendix S1), providing evidence of omnivory in *H. thoracica*. The results match those of Lodge (2000), who recorded invertebrate fragments in 87% of all wild *H. maori* frass that she examined from the Rock and Pillar Range in Otago. Lodge also calculated that invertebrate fragments constituted 10% of all identifiable fragments in her study, similar to the 11% seen here. We observed considerable variance in the number of invertebrate fragments per pellet, suggesting that omnivory could be opportunistic.

Omnivorous insects from both herbivorous (such as Orthopteran) and predatory (such as spider) lineages can acquire a large percentage of their nitrogen needs by eating other insects (e.g., Denno & Fagan, 2003). If this applies to tree wētā, it could explain Dewhurst's (2012) finding that leaf N concentration did not predict plant foraging preference in tree wētā. We found that invertebrate fragment density in frass differed significantly between upper hillslope and terrace habitat zones in this study. This could indicate different foraging strategies by *H. thoracica* in these locations. We did not measure invertebrate abundance in the habitat zones, but both the terrace and mid‐hillslope habitat zones have a diverse shrub vegetation, whereas the upper hillslope has little ground cover in many areas.

The data indicate that *H. thoracica* discriminate between available food sources and are selective foragers (Appendix S1) and that lipid‐rich plants tend to be consumed more frequently than predicted. We found that the plants that scored neutral to positive electivity rankings on Ivlev's Electivity Index had a higher mean concentration of lipids in their leaves than other available plants that scored strongly negative electivity rankings (Figure 3). The preferred group had an average hexane‐soluble lipid concentration somewhere between 0.07 and 1.57 mg/g higher than the nonpreferred group, with 95% confidence. In a closely related subalpine species, *H. maori*, plant species with higher concentrations of lipids in their leaves were eaten more frequently than other plants in the same area of the Rock and Pillar ranges (Joyce, 2002; Lodge, 2000; Wilson, 2004). Data that specifically include analyses of distance travelled from wētā cavities to lipid‐rich and other food sources, as well as frass fragment analysis, would be useful to further understand the intake of lipid‐rich food sources that are essential for reproduction and growth.

A range of plant species were consumed more frequently than predicted by their availability. Ivlev's Electivity Index indicated that plants occurring at multiple sites demonstrate similar electivity. Examples include *D. dacrydioides, P. totara*,* K. ericoides,* and *Melicytus* sp., which all show positive electivity, in multiple locations. However, *H. thoracica* also utilizes other species in environments with different floral communities. For example, on the terrace where *K. ericoides* is not present, *H. thoracica* utilized *P. corymbosa*. *Pennantia corymbosa* is in turn not available to upper hillslope *H. thoracica*, who instead utilize *C. serratus* (which is not available to either the terrace or mid‐hillslope populations). *H. thoracica* can therefore utilize the same food sources in a range of habitats, but they can also exploit new species they encounter in the absence of these species.

*Hemideina thoracica* demonstrated both positive preference and aversion toward plant species in the habitat. For example, *K. ericoides* showed high electivity in both of the habitats where it was present, and *D. dacrydioides* and *Melicytus* sp. showed high electivity in two of the three habitats where they were present. In comparison, the introduced privet *L. sinense* was common, but appeared to be a nonpreferred plant food at all three habitat zones. Interestingly, 14 of 18 *H. thoracica* captured on the terrace were extracted from holes in live *L. sinense*, which had an adjusted ground cover of 24.56% in that habitat, but *L. sinsense* made up only 3.85% of all fecal fragments identified in frass from the terrace. Other plants that were common, but almost completely absent from the frass, were lycopods, many ferns and angiosperms such as grasses, *Beilschmiedia tawa, Litsea calicaris, Myrsine australis*,* Metrosideros diffusa*, and *Geniostoma ligustrifolium*. For example, the introduced grass *Microstegium vimineum* and the clubmoss *Selaginella* were common ground cover plants, but were completely absent from the frass. Similarly, fern species were common in all habitats, but <40% of the individual frass pellets contained fern fragments, and only a single pellet was found to be >1% fern fragments by composition. Differences in digestibility between plant species may lead to underestimation of species such as ferns, however, and laboratory trials might help further identify palatability and preferences for these species.

In this study, the reconnaissance plot sampling method was used to transform tree cover in an approximation of leaf fall area. A limitation of this technique is the fact that tree species vary in the timing of leaf fall and environmental factors affect the final distribution of the leaves. Because of this, the recce technique may not necessarily reflect what is actually available to animals foraging on the ground in a given plot. In any future fragment analyses, rather than standard recce plots, it would be interesting to conduct litter plots on the ground to identify the composition of the leaves available there.

Our findings support the hypothesis that *H. thoracica* forage selectively in the wild and that lipid concentration may contribute to this selectivity. However, although *H. thoracica* favoured some lipid‐rich plant species, some common plants which had relatively high lipid concentrations, such as *D. cupressinum* and *C. dealbata*, were not found in frass. It is likely that other factors not measured here influence food choices. For example, toxic plant secondary metabolites (PSMs) strongly influence the feeding behavior of most leaf‐eating generalists (Behmer et al., 2002; Nersesian et al., 2011, 2012; Raubenheimer, 1992; Raubenheimer & Simpson, 1990; Wiggins, MacArthur, Davies, & McLean, 2006).

Biomechanical properties of leaves and other plant tissues may also be important in food source selection for generalists. Leaf structural defences are a critical biomechanical barrier for leaf‐chewing insects (Clissold, 2007; Clissold, Sanson, Read, & Simpson, 2009; Kitajima & Poorter, 2008, 2010) and for many species, temporal and spatial patterns of foraging are strongly correlated with leaf structural composition (Casher, 1996; Kitajima & Poorter, 2008, 2010; Lowman & Box, 1983; Ohmart & Edwards, 1991). Both the potential effects of metabolites and biomechanical leaf traits on tree wētā foraging remain to be investigated.

In summary, this study has demonstrated that *H. thoracica* selectively forage on a range of plant species and that invertebrates and fruit and seeds are included in their dietary choices. Lipid content may contribute to food choice, but further work is required to understand preferences for lipid and nitrogen content preferences in plant material. *Hemideina thoracica* individuals forage in a nutritionally heterogeneous environment and experience a wide variety of nutrients and secondary metabolites in the plants they encounter. Further field studies still need to be performed on this and other species of tree wētā, in conjunction with controlled laboratory experiments, in order to gain a greater understanding of the roles of both chemical and biomechanical factors in the foraging behavior of these animals.

## CONFLICT OF INTEREST

None declared.

## AUTHOR CONTRIBUTIONS

MB, CECG, and PMW designed the project. MB conducted all field and most experimental work with advice from PMW; CECG supervised the lipid analyses. MB and SM led the data analyses and all authors interpreted data. MB, CECG, SM, and PMW wrote the manuscript.

## References

[ece33763-bib-0001] Ali, J. G. , & Agrawal, A. A. (2012). Specialist versus generalist insect herbivores and plant defence. Trends in Plant Science, 17(5), 293–302. https://doi.org/10.1016/j.tplants.2012.02.006 22425020 10.1016/j.tplants.2012.02.006

[ece33763-bib-0002] Beenakkers, A. T. , Van der Horst, D. J. , & Van Marrewijk, W. J. A. (1981). Role of lipids in energy metabolism In Downer R. G. H. (Ed.), Energy metabolism in insects (pp. 53–100). New York, NY: Springer https://doi.org/10.1007/978-1-4615-9221-1

[ece33763-bib-0003] Behmer, S. T. (2009). Insect herbivore nutrient regulation. Annual Review of Entomology, 54, 165–187. https://doi.org/10.1146/annurev.ento.54.110807.090537 10.1146/annurev.ento.54.110807.090537 18764740

[ece33763-bib-0004] Behmer, S. T. , Simpson, S. J. , & Raubenheimer, D. (2002). Herbivore foraging in chemically heterogeneous environments: Nutrients and secondary metabolites. Ecology, 83, 2489–2501. https://doi.org/10.1890/0012-9658(2002)083[2489:HFICHE]2.0.CO;2

[ece33763-bib-0005] Bekhuis, P. D. B. , De Jong, C. B. , & Prins, H. H. T. (2008). Diet selection and density estimates of forest buffalo in Camo‐Ma'an National Park, Cameroon. African Journal of Ecology, 46, 668–675. https://doi.org/10.1111/j.1365-2028.2008.00956.x

[ece33763-bib-0006] Bolnick, D. I. , Svanbäck, R. , Fordyce, J. A. , Yang, L. H. , Davis, J. M. , Hulsey, C. D. , & Forister, M. L. (2002). The ecology of individuals: Incidence and implications of individual specialization. The American Naturalist, 161, 1–28. 10.1086/343878 12650459

[ece33763-bib-0007] Casher, L. E. (1996). Leaf toughness *Quercus agrifolia* and its effects on tissue selection by first instars of *Phryganidia californica* (Lepidoptera: Dioptidae) and *Bucculatrix albertiella* (Lepidoptera: Lionetiidae). Annals of the Entomological Society of America, 89, 109–121. https://doi.org/10.1093/aesa/89.1.109

[ece33763-bib-0008] Clissold, F. J. (2007). The biomechanics of chewing and plant fracture: Mechanisms and implications. Advances in Insect Physiology, 43, 317–372. https://doi.org/10.1016/S0065-2806(07)34006-X

[ece33763-bib-0009] Clissold, F. J. , Sanson, G. D. , Read, J. , & Simpson, S. J. (2009). Gross vs. net income: How plant toughness affects performance of an insect herbivore. Ecology, 90, 3393–3405. https://doi.org/10.1890/09-0130.1 20120808 10.1890/09-0130.1

[ece33763-bib-0010] Denno, R. F. , & Fagan, W. F. (2003). Might nitrogen limitation promote omnivory among carnivorous arthropods? Ecology, 84, 2522–2531. https://doi.org/10.1890/02-0370 10.1890/09-2080.1 21058571

[ece33763-bib-0011] Dewhurst, R. (2012). The diet of tree wētā: Natural and captive folivory preferences of Hemideina crassidens and Hemideina thoracica. MSc Thesis, Massey University, Palmerston North, New Zealand. Retrieved from https://mro.massey.ac.nz/handle/10179/4497

[ece33763-bib-0012] Duthie, C. , Gibbs, G. , & Burns, K. C. (2006). Seed dispersal by wētā. Science, 311, 1575 https://doi.org/10.1126/science.1123544 16543452 10.1126/science.1123544

[ece33763-bib-0013] Ferreira‐Diaz, S. , Valente, D. G. , & Abreu, J. M. F. (2003). Comparison between ethanol and hexane for oil extraction from *Quercus suber* L. Fruits. Fats and Oils, 54, 378–383.

[ece33763-bib-0014] Google Earth . (2013). Waingaro Forest Reserve. 37°40’25.74”S and 174°58’21.33”E. Image date 10 May 2013. Accessed: 1 July 2013.

[ece33763-bib-0015] Griffin, M. J. (2011). Wellington tree wētā (Hemideina crassidens) diet and the effect of some of their dietary choices. MSc Thesis, Massey University, Palmerston North, New Zealand. Retrieved from https://mro.massey.ac.nz/bitstream/handle/10179/2893/01_front.pdf

[ece33763-bib-0016] Griffin, M. J. , Morgan‐Richards, M. , & Trewick, S. A. (2011). Is the tree wētā *Hemideina crassidens* an obligate herbivore*?* New Zealand Natural Sciences, 36, 11–19.

[ece33763-bib-0170] Hara, A. , & Radin, N. S. (1978). Lipid extraction of tissues with a low‐toxicity solvent. Analytical Biochemisty, 90, 420–426. https://doi.org/10.1016/0003-2697(78)90046-5 10.1016/0003-2697(78)90046-5 727482

[ece33763-bib-0017] Hothorn, T. , Bretz, F. , & Westfall, P. (2008). Simultaneous inference in general parametric models. Biometrical Journal, 50, 346–363. https://doi.org/10.1002/(ISSN)1521-4036 18481363 10.1002/bimj.200810425

[ece33763-bib-0018] Hurst, J. M. , & Allen, R. B. (2007). A permanent plot method for monitoring indigenous forests – Expanded manual. Unpublished Landcare Research Contract Report LC0708/028. Retrieved from https://nvs.landcareresearch.co.nz/Content/PermanentPlot_FieldProtocols.pdf

[ece33763-bib-0019] Ivlev, V. S. (1961). Experimental ecology of the feeding of fishes. New Haven, CT: Yale University Press.

[ece33763-bib-0020] Joern, A. , & Behmer, S. T. (1997). Importance of dietary nitrogen and carbohydrates to survival, growth, and reproduction in adults of the grasshopper *Ageneotettix deorum* (Orthoptera: Acrididae). Oecologia, 112, 201–208. https://doi.org/10.1007/s004420050301 28307571 10.1007/s004420050301

[ece33763-bib-0021] Jones, C. , & Sanders, M. D. (2005). European hedgehog In King C. M. (Ed.), The handbook of New Zealand mammals (2nd ed., pp. 81–94). Melbourne, Vic: Oxford University Press.

[ece33763-bib-0022] Joyce, S. J. (2002). Survival, longevity, diet and development of mountain stone wētā Hemideina maori in the Rock and Pillar Range, New Zealand. MSc thesis, University of Otago, Dunedin.

[ece33763-bib-0023] King, C. M. , & Murphy, E. C. (2005). Stoat In King C. M. (Ed.), The handbook of New Zealand mammals (2nd ed., pp. 261–286). Melbourne, Vic: Oxford University Press.

[ece33763-bib-0024] Kitajima, K. , & Poorter, L. (2008). Functional basis for resource niche partitioning by tropical trees In Carson W. P., & Schnitzer S. A. (Eds.), Tropical forest community ecology (pp. 172–188). Chichester, UK: Blackwell Science.

[ece33763-bib-0025] Kitajima, K. , & Poorter, L. (2010). Tissue‐level leaf toughness, but not lamina thickness predicts sapling leaf lifespan and shade tolerance of tropical trees. New Phytologist, 186, 708–721. https://doi.org/10.1111/j.1469-8137.2010.03212.x 20298481 10.1111/j.1469-8137.2010.03212.x

[ece33763-bib-0026] Lee, K. P. , Cory, J. S. , Wilson, K. , Raubenheimer, D. , & Simpson, S. J. (2006). Flexible diet choice offsets protein costs of pathogen resistance in a caterpillar. Proceedings of the Royal Society B: Biological Sciences, 273, 823–829. https://doi.org/10.1098/rspb.2005.3385 16618675 10.1098/rspb.2005.3385 PMC1560230

[ece33763-bib-0027] Lee‐Yaw, J. A. , Kharouba, H. M. , Bontrager, M. , Mahoney, C. , Csergo, M. A. , Noreen, A. M. E. , … Angert, A. L. (2016). A synthesis of transplant experiments and ecological niche models suggests that range limits are often niche limits. Ecology Letters, 19, 710–722. https://doi.org/10.1111/ele.12604 27111656 10.1111/ele.12604

[ece33763-bib-0028] Leisnham, P. T. , Cameron, C. , & Jamieson, I. G. (2003). Life cycle, survival rates, and longevity of an alpine tree wētā *Hemideina maori* (Orthoptera: Anastostomatidae) determined using mark‐recapture analysis. New Zealand Journal of Ecology, 27(2), 191–200.

[ece33763-bib-0029] Lodge, R. L. S. (2000). In the poo: The diet of the alpine wētā (Hemideina maori) from the Rock and Pillar Range, Central Otago, New Zealand. Diploma of Science dissertation, University of Otago, Dunedin.

[ece33763-bib-0030] Lowman, M. D. , & Box, J. D. (1983). Variation in leaf toughness and phenolic content among 5 species of Australian rain‐forest trees. Australian Journal of Ecology, 8, 17–25. https://doi.org/10.1111/j.1442-9993.1983.tb01515.x

[ece33763-bib-0031] Mayntz, D. , Raubenheimer, D. , Salomon, M. , Toft, S. , & Simpson, S. J. (2005). Nutrient‐specific foraging in invertebrate predators. Science, 307, 111–112. https://doi.org/10.1126/science.1105493 15637278 10.1126/science.1105493

[ece33763-bib-0032] Mayntz, D. , & Toft, S. (2001). Nutrient composition of the prey's diet affects growth and survivorship of a generalist predator. Oecologia, 127, 207–213. https://doi.org/10.1007/s004420000591 24577651 10.1007/s004420000591

[ece33763-bib-0033] Mirams, R. V. (1956). Aspects of the natural regeneration of the kauri (*Agathis australis* Salisb). Transactions of the Royal Society of New Zealand, 84, 661–680.

[ece33763-bib-0034] Moller, H. (1985). Tree wetas (*Hemideina crassicruris*)(Orthoptera: Stenopelmatidae) of Stephens Island, Cook Strait. New Zealand Journal of Zoology, 12, 55–69. https://doi.org/10.1080/03014223.1985.10428265

[ece33763-bib-0035] Nersesian, C. L. , Banks, P. B. , & McArthur, C. (2011). Titrating the cost of plant toxins against predators: Determining the tipping point for foraging herbivores. Journal of Animal Ecology, 80, 753–760. https://doi.org/10.1111/j.1365-2656.2011.01822.x 21366564 10.1111/j.1365-2656.2011.01822.x

[ece33763-bib-0036] Nersesian, C. L. , Banks, P. B. , Simpson, S. J. , & McArthur, C. (2012). Mixing nutrients mitigates the intake constraints of a plant toxin in a generalist herbivore. Behavioural Ecology, 23, 879–888. https://doi.org/10.1093/beheco/ars049

[ece33763-bib-0037] Ohmart, C. P. , & Edwards, P. B. (1991). Insect herbivory on eucalyptus. Annual Review of Entomology, 36, 637–657. https://doi.org/10.1146/annurev.en.36.010191.003225

[ece33763-bib-0038] Pulliam, H. R. (1975). Diet optimization with nutrient constraints. American Naturalist, 109, 765–768. https://doi.org/10.1086/283041

[ece33763-bib-0039] R Core Team . (2013). R: A language and environment for statistical computing. Vienna, Austria: R Foundation for Statistical computing Retrieved from http://www.R-project.org/

[ece33763-bib-0040] Rapport, D. J. (1980). Optimal foraging for complementary resources. American Naturalist, 116, 324–346. https://doi.org/10.1086/283631

[ece33763-bib-0041] Raubenheimer, D. (1992). Tannic acid, protein, and digestible carbohydrate: Dietary imbalance and nutritional compensation in locusts. Ecology, 73, 1012–1027. https://doi.org/10.2307/1940176

[ece33763-bib-0042] Raubenheimer, D. , Mayntz, D. , Simpson, S. J. , & Toft, S. (2007). Nutrient‐specific compensation following diapause in a predator: Implications for intraguild predation. Ecology, 88, 2598–2608. https://doi.org/10.1890/07-0012.1 18027762 10.1890/07-0012.1

[ece33763-bib-0043] Raubenheimer, D. , & Simpson, S. J. (1990). The effects of simultaneous variation in protein, digestible carbohydrate, and tannic acid, on the feeding behaviour of larval *Locusta migratoria* (L.) and *Schistocerca gregaria* (Forskal). I. Short term studies. Physiological Entomology, 5, 219–223. https://doi.org/10.1111/j.1365-3032.1990.tb00510.x

[ece33763-bib-0044] Ruscoe, W. A. , & Murphy, E. C. (2005). House mouse In King C. M. (Ed.), The handbook of New Zealand mammals (2nd ed., pp. 204–221). Melbourne, Vic: Oxford University Press.

[ece33763-bib-0045] Schiff, N. M. , Waldbauer, G. P. , & Friedman, S. (1988). Dietary self‐selection for vitamins and lipid by larvae of the corn earworm *Heliothis zea* . Entomologia experimentalis et applicata, 46, 249–256. https://doi.org/10.1111/j.1570-7458.1988.tb01119.x

[ece33763-bib-0046] Simpson, S. J. , Sibly, R. M. , Lee, K. P. , Behmer, S. T. , & Raubenheimer, D. (2004). Optimal foraging when regulating intake of multiple nutrients. Animal Behaviour, 68, 1299–1311. https://doi.org/10.1016/j.anbehav.2004.03.003

[ece33763-bib-0047] Singer, M. S. , Bernays, E. A. , & Carriere, Y. (2002). The interplay between nutrient balancing and toxin dilution in foraging by a generalist insect herbivore. Animal Behaviour, 64, 629–643. https://doi.org/10.1006/anbe.2002.3082

[ece33763-bib-0048] Slansky, F. , & Wheeler, G. S. (1991). Food consumption and utilization responses to dietary dilution with cellulose and water by velvetbean caterpillars *Anticarsia gemmatalis* . Physiological Entomology, 16, 99–116. https://doi.org/10.1111/j.1365-3032.1991.tb00547.x

[ece33763-bib-0049] Strauss, R. E. (1979). Reliability estimates for Ivlev's Electivity Index, the forage ratio, and a proposed linear index of food selection. Transactions of the American Fisheries Society, 108, 344–352. https://doi.org/10.1577/1548-8659(1979)108<344:REFIEI>2.0.CO;2

[ece33763-bib-0050] Townsend, J. A. , Brown, B. , Stringer, I. A. N. , & Potter, M. A. (1997). Distribution, habitat and conservation status of *Hemideina ricta* and *H. femorata* on Banks Peninsula, New Zealand. New Zealand Journal of Ecology, 21, 43–49.

[ece33763-bib-0051] Wehi, P. M. , & Hicks, B. J. (2010). Isotope fractionation in a large herbivorous insect, the Auckland tree wētā. Journal of Insect Physiology, 56, 1877–1882. https://doi.org/10.1016/j.jinsphys.2010.08.005 20709068 10.1016/j.jinsphys.2010.08.005

[ece33763-bib-0052] Wehi, P. M. , Raubenheimer, D. , & Morgan‐Richards, M. (2013). Tolerance for nutrient imbalance in an intermittently feeding herbivorous cricket, the Wellington Tree Weta. PLoS ONE, 8(12), e84641 https://doi.org/10.1371/journal.pone.0084641 24358369 10.1371/journal.pone.0084641 PMC3866171

[ece33763-bib-0053] Wiggins, N. L. , MacArthur, C. , Davies, N. W. , & McLean, S. (2006). Spatial scale of the patchiness of plant poisons: A critical influence on foraging efficiency. Ecology, 87, 2236–2243. https://doi.org/10.1890/0012-9658(2006)87[2236:SSOTPO]2.0.CO;2 16995624 10.1890/0012-9658(2006)87[2236:ssotpo]2.0.co;2

[ece33763-bib-0054] Wilder, S. M. , Norris, M. , Lee, R. W. , Raubenheimer, D. , & Simpson, S. J. (2013). Arthropod food webs become increasingly lipid‐limited at higher trophic levels. Ecology Letters, 16, 895–902. https://doi.org/10.1111/ele.12116 23701046 10.1111/ele.12116

[ece33763-bib-0055] Wilson, C. G. (2004). The roles of melanism in insects, and the diet and distribution of melanic and non‐melanic morphs of the mountain stone wētā Hemideina maori (Orthoptera: Anostostomatidae). MSc Thesis, University of Otago, Dunedin.

[ece33763-bib-0056] Wilson, C. G. , & Jamieson, I. G. (2005). Does melanism influence the diet of the mountain stone wētā *Hemideina maori* (Orthoptera: Anostostomatidae)? New Zealand Journal of Ecology, 29, 149–152.

