# Supplementary figures and images for: Diet selectivity in a terrestrial forest invertebrate, the Auckland tree wētā, across three habitat zones
Source: Ecol Evol. 2018 Feb 1;8(5):2495–503. doi: 10.1002/ece3.3763 (PMC5838035; doi:10.1002/ece3.3763)

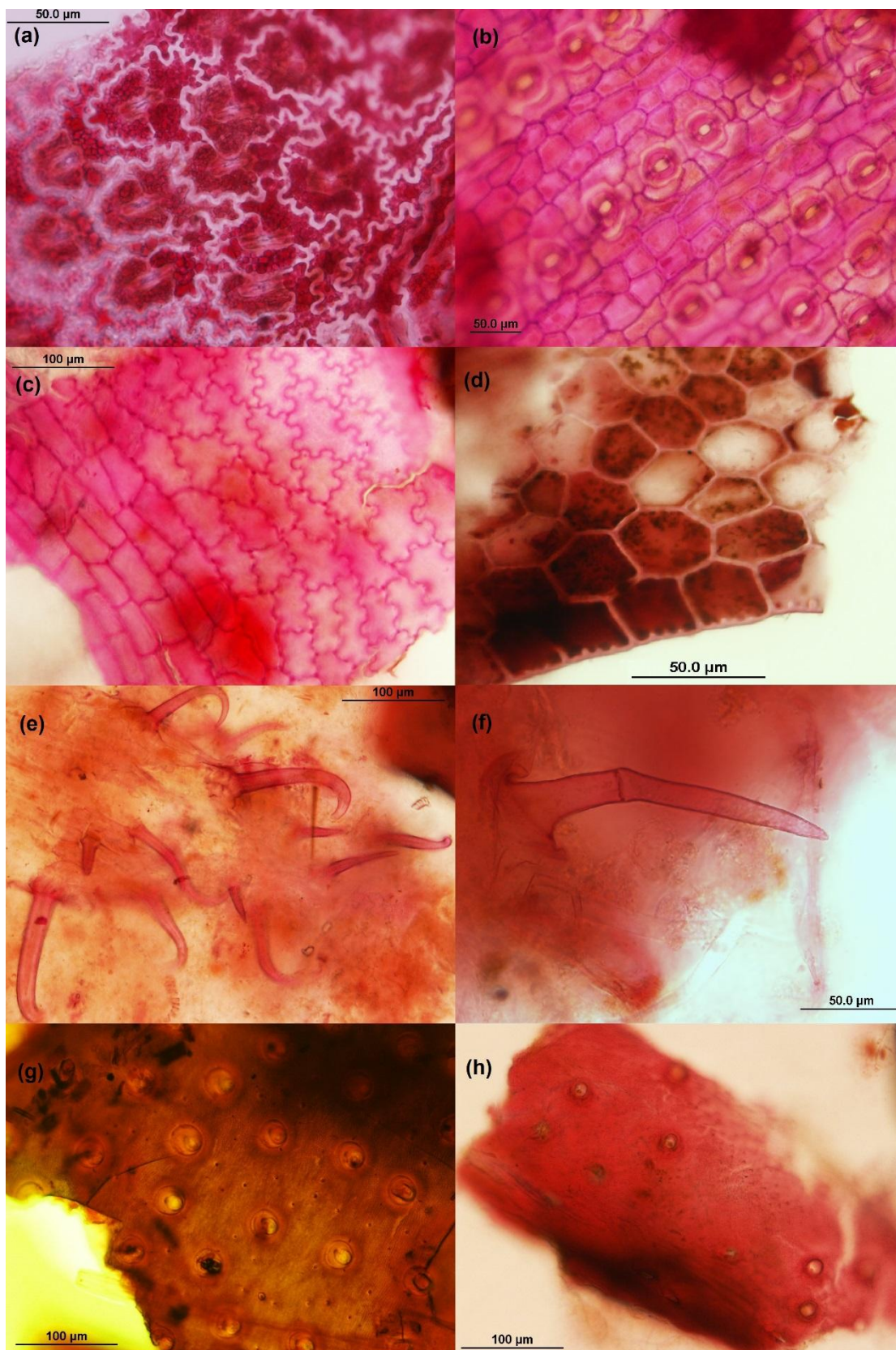

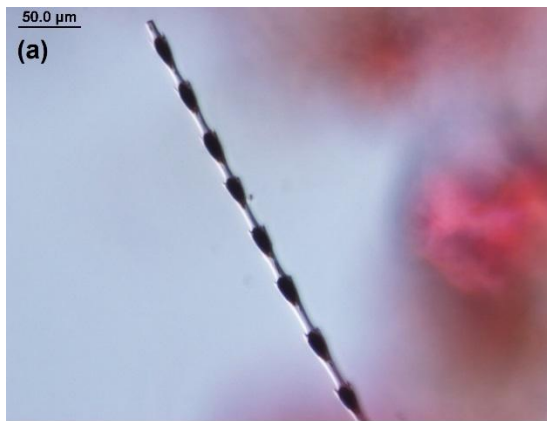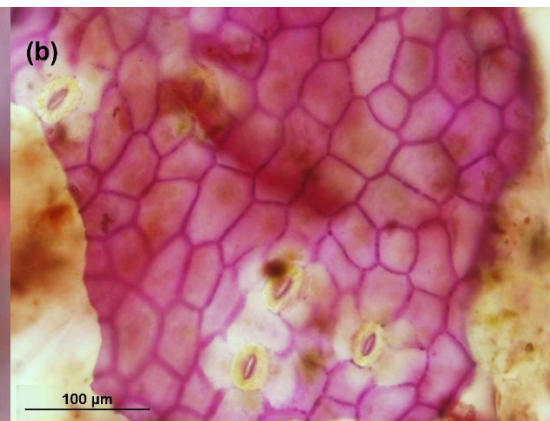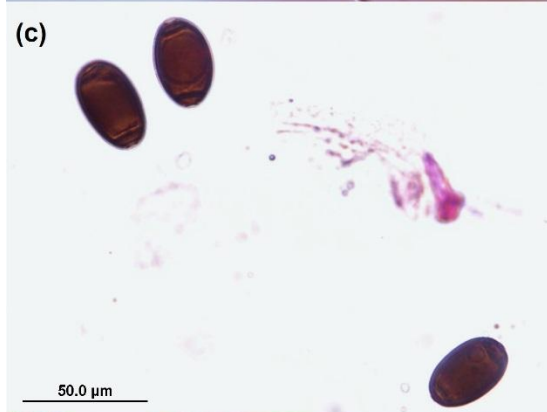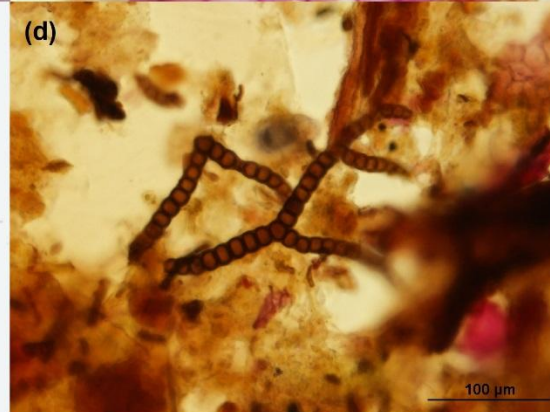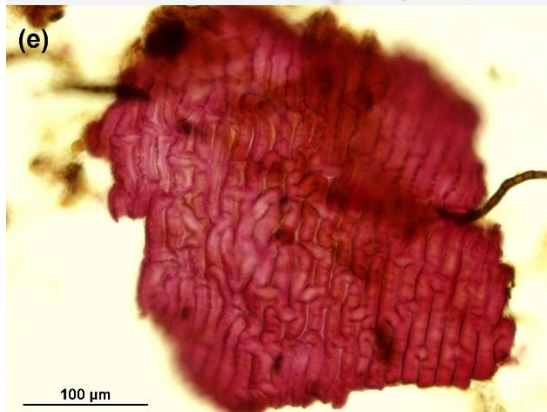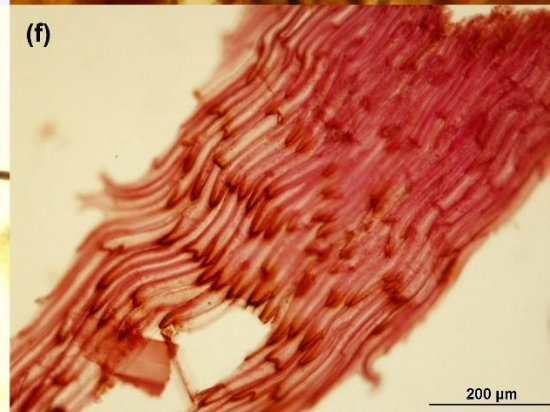

Supplement: Supplementary file 2 [file ECE3-8-2495-s002.pdf]
