# Supplementary material for: Diet selectivity in a terrestrial forest invertebrate, the Auckland tree wētā, across three habitat zones
Source: Ecol Evol. 2018 Feb 1;8(5):2495–503. doi: 10.1002/ece3.3763 (PMC5838035; doi:10.1002/ece3.3763)
Supplement: Supplementary file 3 [file ECE3-8-2495-s003.pdf]

	Wētā ID	Sex	# of frass	Par	Hym	Pyr ser	Cya dea	Dac cup	Dac dac	Pod tot	Pru tax	Cop rot	Kun eri	Lig sin	Myr aus	Mel	Pen cor	Psu cra	Rho sap	Un. 1	Un. 2	Un. 3	Un. 4	Un. 5	Un. 6	Fruit+Seed	Invert	*Sum*
**Terrace**	T2A	M	4	0	1	5	4	30	79	20	148	400		73		44	0		1	0	0					21	2	*822.00*
	T2B	F	3	0	0	1	1	11	85	97	59	23		94		46	343		0	0	84					23	7	*873.00*
	T2C	M	4	15	0	0	0	5	8	0	753	40		3		0	125		0	0	0					182	4	*1120.00*
	T2D	F	3	2	1	0	0	0	0	55	269	13		44		12	202		0	1	244					84	40	*964.00*
	T2E	M	3	0	4	0	0	9	5	0	525	259		81		17	35		0	0	3					37	2	*973.00*
	T3A	M	4	0	0	0	0	22	145	0	439	0		5		4	16		0	0	0					26	4	*661.00*
	T3B	F	3	0	0	0	2	12	7	0	906	125		30		750	5		3	5	1					115	4	*1965.00*
	T3C	M	2	0	0	0	0	5	6	0	159	6		30		124	0		0	0	0					7	4	*341.00*
	T3D	F	3	0	0	0	0	0	502	0	1	1		0		1	0		0	0	0					0	3	*508.00*
	T3E	M	2	0	11	0	12	0	18	350	35	0		0		1	17		0	0	0					394	275	*1102.00*
	T6A	F	4	0	3	0	0	0	290	8	1100	1		0		0	0		0	131	0					498	12	*2040.00*
	T7A	M	4	0	0	0	3	1	1375	0	7	0		4		6	50		0	0	0					1462	527	*3435.00*
	T7B	F	3	0	14	2	1	16	1117	0	28	0		141		1149	6		0	1	0					807	185	*3451.00*
	T7C	F	4	0	2	0	0	0	540	0	328	0		162		70	10		0	2	0					20	8	*1140.00*
	T7D	F	2	0	1	0	0	0	200	0	126	0		8		3	0		0	0	0					1	126	*464.00*
	T10A	M	3	0	0	0	0	0	0	118	463	0		115		0	0		0	191	0					20	19	*926.00*
	Sum		*48*	*17*	*37*	*8*	*23*	*111*	*4377*	*530*	*4883*	*868*		*675*		*2227*	*809*		*4*	*140*	*332*					*3677*	*1203*	*19859.00*
	Percent			*0.08*	*0.18*	*0.04*	*0.11*	*0.53*	*21.00*	*3.11*	*25.64*	*4.16*		*3.79*		*10.68*	*3.88*		*0.02*	*1.59*	*1.59*					*17.73*	*5.86*	*99.70*
**Mid Hillslope**	LS7A	F	2		0		11	0	367	140	230	0	0	2	103	0	0			0		0	0	650		586	14	*2103.00*
	MS4A	M	3		0		0	0	29	0	11	0	0	0	0	11	0			0		0	0	0		865	0	*916.00*
	MS4B	F	3		3		0	0	0	60	0	0	1150	0	0	123	8			1		2000	0	0		71	600	*4016.00*
	MS4C	F	1		0		0	0	3	0	0	0	26	0	1	4	0			0		150	0	0		3	200	*387.00*
	MS4D	M	2		0		0	1	30	420	30	0	0	0	1	1	0			0		0	0	0		978	12	*1473.00*
	MS4E	M	3		0		0	0	29	3	0	12	0	11	3	11	0			0		0	0	0		900	0	*969.00*
	MS4F	F	4		1		0	0	1	93	1	208	0	0	6	236	81			0		0	0	0		163	26	*816.00*
	MS5A	M	3		2		0	32	6	250	3	0	204	0	0	581	0			1		0	0	0		326	21	*1424.00*
	MS5B	F	2		0		0	10	0	428	0	0	0	0	0	0	0			0		1	800	0		251	13	*1503.00*
	MS5C	F	2		0		0	0	0	16	2	0	27	0	0	650	0			0		1	0	0		320	4	*1020.00*
	MS5D	F	2		0		300	0	0	400	259	36	302	0	11	0	0			0		4	0	0		12	17	*1341.00*
	MS5E	F	1		0		1	0	0	10	1	0	0	43	0	0	0			0		0	0	0		1	100	*156.00*
	MS6A	M	2		0		0	167	0	0	0	0	0	0	0	0	0			0		0	0	0		250	167	*584.00*
	Sum		*30*		*6*		*312*	*210*	*465*	*1820*	*537*	*256*	*1709*	*56*	*125*	*1617*	*89*			*2*		*2156*	*800*	*650*		*4726*	*1174*	*16708.00*
	Percent				*0.04*		*1.87*	*1.26*	*2.78*	*10.89*	*3.21*	*1.53*	*10.23*	*0.34*	*0.75*	*9.68*	*0.53*			*0.01*		*12.90*	*4.79*	*3.89*		*28.28*	*7.03*	*99.96*
**Upper Hillslope**	US1A	M	4		1		0			318	1	0	692		2	0		0	0	13			1000		9	229	646	*2591.00*
	US1B	M	3		19		1			52	0	0	405		0	0		0	1	0			0		0	65	203	*674.00*
	US1C	F	3		0		0			989	0	0	1000		0	0		0	0	1			0		0	1	16	*2007.00*
	US2A	M	1		0		0			0	0	0	63		0	0		0	0	0			0		23	0	0	*86.00*
	US2B	F	3		2		1			179	0	0	73		0	0		15	0	5			0		0	114	29	*1229.00*
	US3A	F	3		1		3			160	0	1	0		0	1		0	0	0			0		0	922	750	*1838.00*
	US6A	F	2		0		0			803	0	0	125		0	0		0	0	0			0		0	0	550	*1478.00*
	US6B	M	4		0		0			1653	1	0	188		0	0		0	0	0			7		0	5	256	*456.00*
	US9A	F	3		0		0			415	0	0	0		0	0		0	0	0			0		6	0	0	*421.00*
	US9B	M	4		412		0			473	0	2	0		0	0		0	0	0			0		1125	36	84	*1247.00*
	US10A	M	2		0		1			6	0	177	50		0	0		0	0	0			0		0	100	242	*569.00*
	US10B	M	2		0		0			0	0	0	600		0	0		0	0	1			0		0	450	172	*1223.00*
	US10C	F	2		25		0			302	0	0	300		0	0		0	0	0			0		0	0	281	*908.00*
	US10D	F	4		33		0			102	0	1	1		0	0		0	0	0			0		0	0	700	*837.00*
	Sum		*30*		*435*		*5*			*5042*	*2*	*3*	*2546*		*2*	*1*		*15*	*1*	*19*			*1007*		*1163*	*1372*	*2534*	*12027.00*
	Percent				*2.66*		*0.03*			*29.47*	*0.01*	*0.98*	*18.90*		*0.01*	*0.01*		*0.08*	*0.01*	*0.11*			*5.44*		*9.67*	*10.39*	*21.24*	*66.83*
