# Supplementary material for: Diet selectivity in a terrestrial forest invertebrate, the Auckland tree wētā, across three habitat zones
Source: Ecol Evol. 2018 Feb 1;8(5):2495–503. doi: 10.1002/ece3.3763 (PMC5838035; doi:10.1002/ece3.3763)
Supplement: Supplementary file 1 [file ECE3-8-2495-s001.pdf]

		Terrace			Mid Hillslope			Upper Hillslope		
**Main Group**										
Family	Genus/Species	AC	F	IEI	AC	F	IEI	AC	F	IEI
**Lichens**										
Parmeliaceae	*Parmelia* sp.		0.08							
**Fern Leaf**										
Cyatheaceae	*Cyathea dealbata*	4.34	0.11	1.00	16.96	1.87	-0.80	9.28	0.03	0.99
Hymenophyllaceae	*Hymenophyllum flabellatum*		0.18		0.18	0.04	0.67	0.19	2.66	0.87
Polypodiaceae	*Pyrosia eleagnifolia*	0.38	0.04	0.82				4.00		-1.00
**Gymnosperm Leaf**										
Podocarpaceae	*Dacrydium cupressinum*	3.78	0.53	0.75	2.68	1.26	0.36			
	Dacrycarpus dacrydioides	6.61	21.00	0.52	3.57	2.78	0.12			
	Podocarpus totara	4.72	3.11	0.21	5.36	10.89	0.34	19.12	29.47	0.21
	Prumnopitys taxifolia	20.78	25.64	0.10	7.14	3.21	0.38	0.22	0.01	0.91
**Angiosperm Leaf**										
Rousseaceae	*Carpodetus serratus*				1.79		-1.00	1.86	4.38	0.40
Rubiaceae	*Coprosma rotundifolia*	8.69	4.16	0.35	3.61	1.53	-0.40	2.78	0.98	-0.48
Myrtaceae	*Kunzea ericoides*				3.57	10.23	0.48	9.28	18.90	0.34
Oleaceae	*Ligustrum sinense* (invasive)	24.56	3.79	0.73	4.64	0.34	0.87	3.60		-1.00
Violaceae	*Melicytus* sp.	4.72	10.68	0.39	5.53	9.68	0.27	5.94	0.01	-1.00
Myrsinaceae	*Myrsine australis*				1.96	0.75	0.45	2.52	0.01	0.99
Pennantiaceae	*Pennantia corymbosa*	2.08	3.88	0.30	2.32	0.53	0.63			
Araliaceae	*Pseudopanax crassifolium*	0.08		-1.00	1.00		-1.00	2.04	0.08	0.92
Arecaceae	*Rhopalostylus sapida*	2.72	0.02	0.99	1.10		-1.00	0.71	0.01	0.98
Fruit and seeds			17.73			28.28			10.39	
Invertebrates			5.86			7.03			21.24	
Unidentified	Unidentified 1		1.59			0.01			0.11	
	Unidentified 2		1.59							
	Unidentified 3					12.90				
	Unidentified 4					4.79			5.44	
	Unidentified 5					3.89				
	Unidentified 6								6.29	
